# Quantitative Predictions of the Thermal Conductivity in Transition Metal Dichalcogenides: Impact of Point Defects in MoS_2_ and WS_2_ Monolayers

**DOI:** 10.1021/acs.jpcc.3c06820

**Published:** 2024-01-18

**Authors:** Srinivasan Mahendran, Jesús Carrete, Andreas Isacsson, Georg K. H. Madsen, Paul Erhart

**Affiliations:** † Department of Physics, Chalmers University of Technology, SE-41296 Gothenburg, Sweden; ‡ Instituto de Nanociencia y Materiales de Aragón (INMA), CSIC-Universidad de Zaragoza, E-50009 Zaragoza, Spain; § Institute of Materials Chemistry, TU Wien, A-1060 Vienna, Austria

## Abstract

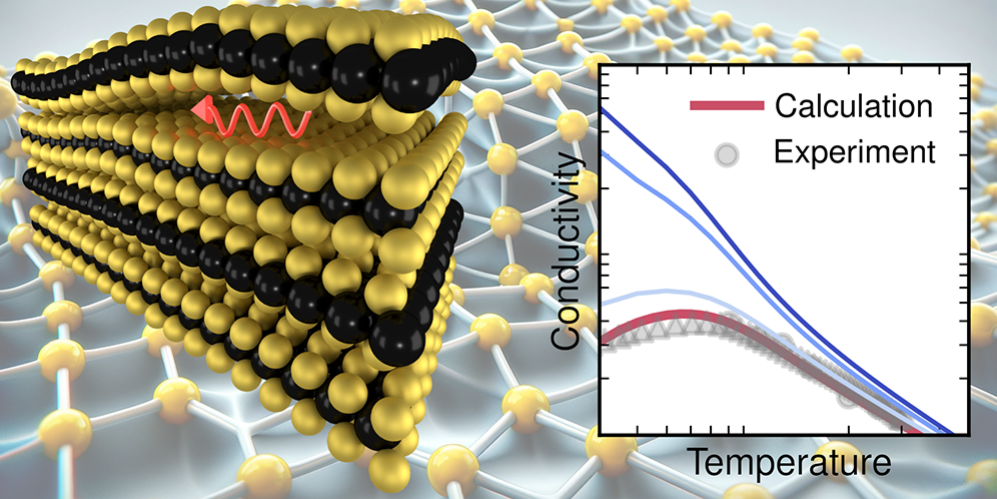

Transition metal dichalcogenides are investigated for various applications at the nanoscale because of their unique combination of properties and dimensionality. For many of the anticipated applications, heat conduction plays an important role. At the same time, these materials often contain relatively large amounts of point defects. Here, we provide a systematic analysis of the impact of intrinsic and selected extrinsic defects on the lattice thermal conductivity of MoS_2_ and WS_2_ monolayers. We combine Boltzmann transport theory and Green’s function-based **T**-matrix approach for the calculation of scattering rates. The force constants for the defect configurations are obtained from density functional theory calculations via a regression approach, which allows us to sample a rather large number of defects at a moderate computational cost and to systematically enforce both the translational and rotational acoustic sum rules. The calculated lattice thermal conductivity is in quantitative agreement with the experimental data for heat transport and defect concentrations for both MoS_2_ and WS_2_. Crucially, this demonstrates that the strong deviation from a 1/*T* temperature dependence of the lattice thermal conductivity observed experimentally can be fully explained by the presence of point defects. We furthermore predict the scattering strengths of the intrinsic defects to decrease in the sequence V_Mo_ ≈ V_2S_^=^ > V_2S_^⊥^ > V_S_ > S_ad_ in both materials, while the scattering rates for the extrinsic (adatom) defects decrease with increasing mass such that Li_ad_ > Na_ad_ > K_ad_. Compared with earlier work, we find that both intrinsic and extrinsic adatoms are relatively weak scatterers. We attribute this difference to the treatment of the translational and rotational acoustic sum rules, which, if not enforced, can lead to spurious contributions in the zero-frequency limit.

## 1 Introduction

Transition metal dichalcogenides (TMDs) have emerged as fascinating materials in the fields of nanotechnology, electronics, and optoelectronics due to their quasi-two-dimensional (2D) structure and associated properties. Notable examples are molybdenum disulfide (MoS_2_) and tungsten disulfide (WS_2_). These 2D semiconducting compounds possess unique electronic, optical, and mechanical characteristics distinct from those of their bulk counterparts. The distinctive characteristics arise from their ultrathin atomic layers, which makes them promising candidates for the development of next-generation nanodevices, including transistors, sensors, photodetectors, and catalysts.^1−5^

The ability to efficiently conduct heat or, reciprocally, to impede its transport can greatly impact the reliability, power dissipation, and thermal management of these materials in various applications, ranging from microelectronics to energy conversion systems.^6^ It is therefore important to quantify the thermal conductivity and specifically the lattice thermal conductivity (LTC) in these materials^7,8^ and understand the limiting factors. In this context, calculations can be tremendously useful, in particular, when rooted in an ab initio framework. Yet, such calculations based on Boltzmann transport theory or Green–Kubo relations typically overestimate the LTC and yield a stronger temperature dependence than observed experimentally.^9,10^ The deviation can be accounted for by invoking a heuristic, semiempirical boundary scattering term, which obfuscates the underlying reason for the discrepancy.

For fully periodic, three-dimensional materials such as SiC^11^ and GaAs,^12^ it has been shown that the inclusion of point defect scattering is crucial for obtaining quantitative agreement with experimental measurements of the LTC. Because of the two-dimensional nature of TMDs such as MoS_2_ and WS_2_, it is possible to image defects directly, e.g., via scanning tunneling microscopy (STM) or scanning transmission electron microscopy (STEM), which points to the S vacancy as the most abundant intrinsic defect.^13−18^ These investigations have also shown the defect population to be sensitive to the synthesis conditions^14^ and to exhibit considerable spatial variations.^15,19,20^

While the majority of LTC research on TMDs so far has focused on phonon–phonon and isotope scattering in computational analyses, a few studies have also considered scattering by point defects and impurities.^21−24^ While these investigations clearly demonstrated the potential impact of defects, they are limited in scope, either because of the reliance on semiempirical ingredients or because of the limited number of defect types considered (in particular, extrinsic defects).

Methods based on the Boltzmann transport formalism afford very detailed insight into phonon scattering, flexibility when it comes to defect concentrations, and, when combined with *ab initio* theory, proven predictive value. However, in the case of quasi-2D systems very careful attention must be paid to the postprocessing of the *ab initio* calculations to remove the effects of boundary conditions that violate the symmetries of free space.^25^ With that in mind, here, we provide a physically founded, systematic analysis of the impact of intrinsic and selected extrinsic defects on the LTC of MoS_2_ and WS_2_ monolayers. This allows us to provide a quantitative description of the measured LTC without resorting to semiempirical models and/or parameters.

## 2 Methodology

### 2.1 Lattice Thermal Conductivity

The phononic contribution to the LTC can be calculated by solving the Boltzmann transport equation (BTE). In this work the BTE is solved under the relaxation time approximation (RTA), which yields the following expression for the LTC:
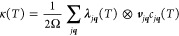
1Here, Ω is the volume of unit cell while **λ**_*jq*_, **υ**_*j****q***_, and *c*_*j****q***_ are the phonon mean free path (MFP) (interpreted as a vector), group velocity, and specific heat capacity of mode *j* with momentum vector ***q***, respectively. The MFP **λ**_*jq*_ = τ_*jq*_(*T*)**υ**_*jq*_ is proportional to the relaxation time τ_*j****q***_. In this work the inverse of the relaxation time, i.e., the total scattering rate τ_*j****q***_^–1^, is calculated using Matthiesen’s rule by adding the contributions from phonon–phonon (ph–ph), isotope (iso), and defect scattering (def):
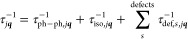
In this work the phonon–phonon scattering rate, τ_ph–ph,*j****q***_^–1^, associated with the anharmonicity of the material is evaluated considering three-phonon processes. The τ_iso,*j****q***_^–1^ term represents isotopic mass disorder and gives rise to a temperature-independent scattering rate. The calculation of the defect scattering rates is described in the next section. For comparison, we also consider a model for boundary scattering given by
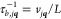
2where *L* is the characteristic length of the structural homogeneity of the material.

### 2.2 Phonon Scattering by Defects

The defect scattering rates can be obtained by using the optical theorem:
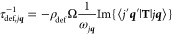
Here, ρ_def_ is the volumetric defect concentration and ω_*j****q***_ is the angular phonon frequency. The **T**-matrix links the phonon wave functions of the ideal and defect-laden systems through the relation
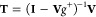
where *g*^+^ is the retarded Green’s function of the ideal structure while **V** represents the perturbation connecting the ideal and the defect-laden systems, which can be decomposed as
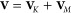
Here, the diagonal matrix **V**_*M*_ describes changes in mass, with elements
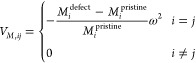
while **V**_*K*_ captures changes in the constants (FCs) and is given by
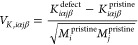
Here, *K*_*i*α*j*β_ is the FC matrix where *i* and *j* are atomic indices while α and β denote Cartesian indices.

In the cases of vacancies and adatoms, the creation of a defect can be conceptualized as the connection or disconnection of a subset of atoms to or from the bulk system. The corresponding FC are zero in the pristine system for adatoms and in the defect-laden system for vacancies, but the atoms themselves are not replaced. The value of the mass perturbation becomes irrelevant and can be taken as zero. To successfully implement this idea for vacancies, the Green’s function computed for the pristine system has to be augmented with a block corresponding to a free atom.

We use this Green’s-function-based **T**-matrix approach as implemented in the almaBTE package,^26^ where the linear tetrahedron method^27,28^ is used to calculate the Green’s function of the ideal structure for each value of the incident phonon frequency. This software package has previously been successful in computing thermal transport properties of solids in the presence of varying concentrations of defects, achieving good agreement with experiment.^11,12^

### 2.3 Computational Details

All structures and FC were obtained from density functional theory (DFT) calculations that were performed using the projector augmented-wave method^29^ as implemented in the Vienna Ab-initio Simulation Package (VASP).^30,31^ The exchange-correlation contribution was represented using the van der Waals density functional with consistent exchange (vdW-DFcx) method,^32,33^ which has been previously shown to provide an excellent description for TMDs.^9,34^ All calculations were performed using a plane-wave energy cutoff of 260 eV and Gaussian smearing with a width of 0.1 eV. Projection operators were evaluated in reciprocal space, and a finer support grid was employed during the calculation of the forces to improve the numerical accuracy of the latter. For the primitive cells the Brillouin zone was sampled using a Γ-centered 12 × 12 × 1 grid. For the representation of defect structures as well as the calculation of FC, we used supercells comprising 8 × 8 × 1 primitive cells (192 ± 1 atoms); their Brillouin zone was sampled using a Γ-centered 2 × 2 × 1 grid. A vacuum layer of at least 27 Å was introduced along the axis perpendicular to the monolayer to avoid interactions between periodic images of these quasi-2D systems. However, all values of the thermal conductivities are given for conventional thicknesses of 6.15 Å for MoS_2_ and 6.18 Å for WS_2_.^9^

The large defect-laden supercells, combined with their low symmetry, make it computationally expensive to evaluate the FC through a systematic enumeration of the displacements. For example, calculation of the FC for a sulfur vacancy would require 1146 separate DFT calculations using a direct finite-displacement approach. We therefore resort to a regression approach^35,36^ based on recursive feature elimination via scikit-learn(37) and the hiphive package.^36^ This approach allows one to reduce the number of DFT calculations by 1–2 orders of magnitude depending on cell size.^38^

To obtain second-order FC for the different defect configurations, we generated structures with random displacements sampled from a Gaussian distribution with a standard deviation of 0.01 Å. We then computed the forces for these reference structures via DFT (see above) and used them to construct FC models, splitting them into a training set (75% of the available data) and a validation set (25% of the data). To optimize the quality of the FC extraction, we performed convergence studies with respect to the cutoff imposed on pair interactions (which sets the number of free parameters) and the number of structures included in the training procedure. The FC models also include a small number of third-order terms according to a triplet cutoff of 3 Å, as this has been found to improve the quality of the second-order FC by avoiding the spurious inclusion of small anharmonic contributions.^38^

**Figure 1:**
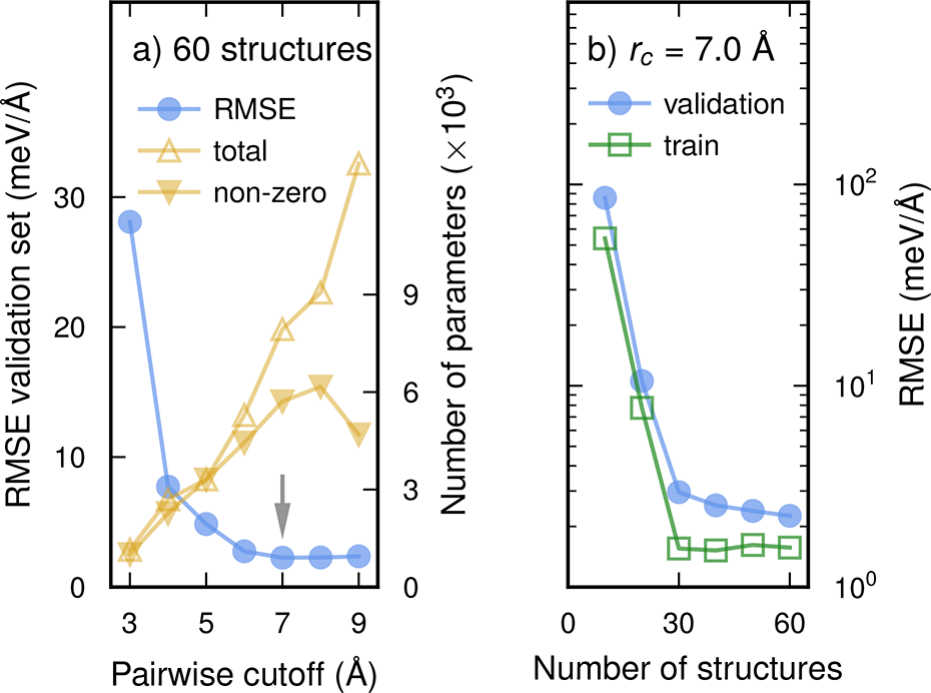
Extraction of force constants for defects. (a) Convergence of the root-mean-square error (RMSE) with the cutoff for pair interactions using a fixed number of reference structures for the sulfur vacancy (V_S_) in MoS_2_. As the cutoff increases, the total number of parameters increases as well, yet the number of nonzero parameters (as well as the RMSE) settle for a cutoff of 7 Å and larger. (b) Convergence with the number of reference structures using a pairwise cutoff of 7 Å.

Using the sulfur vacancy (V_S_) in MoS_2_ as an illustrating example, it can be seen that with a fixed reference set size comprising 60 structures, the root-mean-square error (RMSE) over the validation set quickly drops with increasing pair cutoff and then levels off at 3 meV Å^–1^ for a cutoff of 7 Å (Figure 1a). Increasing the cutoff further does not yield a further reduction, and the number of nonzero parameters remains approximately at the same level.

Next, we demonstrate convergence with respect to the number of structures included in the reference set for a fixed pairwise cutoff of 7 Å (Figure 1b). The RMSE for the validation set is already very low at 30 structures, suggesting that one could reduce the number of configurations for which DFT calculations have to be performed by a factor of approximately 50. Here, we used, however, a more conservative number of 60 configurations, which still leads to very substantial savings in computational effort. The convergence analysis for other defects yields practically identical results, so we proceeded with a pairwise cutoff of 7 Å and reference sets of 60 structures for all defects and both materials (Table S1).

To obtain the second-order FC for MoS_2_ and WS_2_, we generated rattled 20 structures for each material based on the primitive 3-atom unit cell with an average displacement amplitude of 0.2 Å. The numerical errors associated with DFT calculations often lead to a failure to recover the quadratic dispersion of the lowest transverse acoustic mode, which is a hallmark of two-dimensional materials. Such artifacts can have a dramatic effect on the thermal conductivity.^25^ It is therefore important to impose not only the translational but also the rotational acoustic sum rules. In the present work, all sum rules are efficiently enforced via regularization using the hiphive package,^36^ so that even sets of interatomic FC for the defect-laden structures satisfy the symmetries of free space.

The third-order force constants are only needed for the perfects system and were calculated using a systematic finite-displacement approach as implemented in the thirdorder script included with the ShengBTE package.^39^ We included interactions up to the seventh nearest neighbors, which yield cutoffs of 7.08 and 7.09 Å for MoS_2_ and WS_2_, respectively.

To calculate the thermal conductivity, we integrated over the Brillouin zone employing an 83 × 83 × 1 regular Γ-centered grid. To avoid introducing artifacts in the bands through the use of the linear tetrahedron method, the calculation of the Green’s function is performed on a denser 103 × 103 × 1 grid. Note that 83 and 103 are coprime numbers, so the two grids do not share any points other than Γ.

## 3 Results

**Figure 2:**
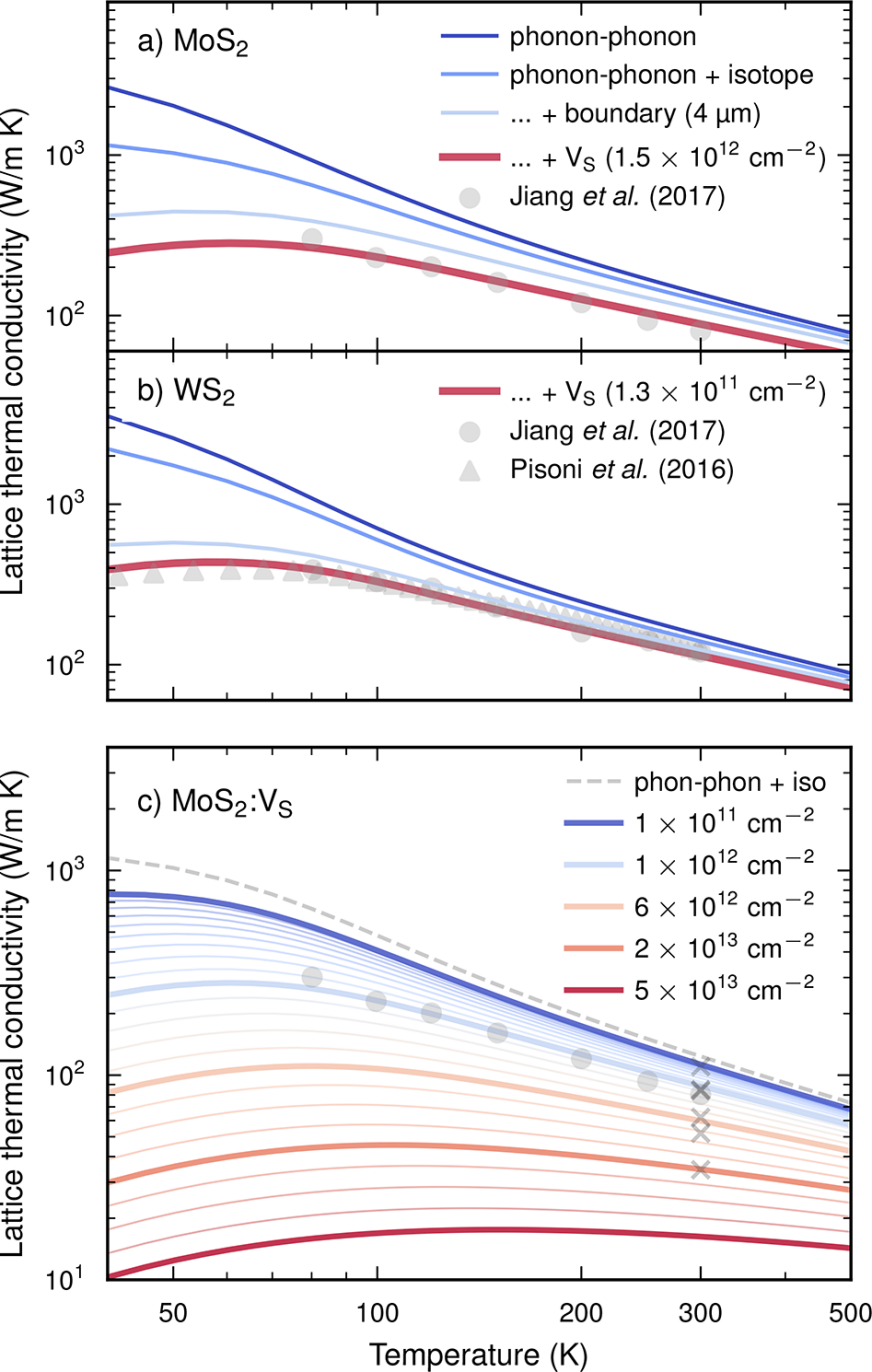
Lattice thermal conductivity of (a, c) MoS_2_ and (b) WS_2_ from calculations accounting for different scattering mechanisms including scattering by sulfur vacancies (*V*_S_) at a fixed concentration (lines) and experiment (symbols). (c) Lattice thermal conductivity of MoS_2_ in the presence of S vacancies over a range of defect concentrations. Temperature-dependent experimental data from Jiang et al. (circles; ref (8)) and Pisoni et al. (triangles; ref (7)). Additional experimental data from refs (45−49) are shown by crosses in (c).

When considering phonon–phonon scattering only, one obtains a LTC that notably overestimates the experimental data^8^ (Figure 2a,b), in particular at lower temperatures; one also observes a more pronounced temperature dependence. While including isotopic mass-variance scattering lowers the LTC somewhat, the prediction is still far from the experimentally measured values.

A semiempirical way to account for the difference is to introduce a boundary scattering term, which introduces an intrinsic length scale that caps the mean free paths of the phonons. This is illustrated here using the length parameter of 4 μm used in ref (9) (light-blue lines in Figure 2a,b). This value is considerably smaller than typical grain sizes in the samples used for comparison here, and while it enables a fit to the data, it fails to provide deeper physical insight.

It is well established that point-defect concentrations in TMDs tend to be rather high, with densities up to 10^13^ cm^–2^.^5,13−20,40,41^ This raises the question of whether defects can quantitatively account for the gap between prediction and measurement, and if so, how effective are different defects with respect to phonon scattering. To answer these questions, we computed the scattering rates for a selection of intrinsic and extrinsic defects, focusing on defects that have been observed experimentally^7,14,17,18^ and/or appear thermodynamically plausible based on first-principles calculations of defect formation energies.^42^ Among the intrinsic defects, we included the Mo (V_Mo_), W (V_W_), and S monovacancies (V_S_), the in-plane (V_2S_^=^) and out-of-plane S divacancies (V_2S_^⊥^), and the S adatom (S_ad_). According to DFT calculations these defects adoptthe neutral charge state for most electron chemical potentials.^43,44^ We therefore restricted ourselves to the neutral charge state.

Among the extrinsic defects, we considered adatoms of Li, Na, and K in the case of MoS_2_ and of Na in WS_2_ as representatives of impurities introduced during exfoliation but also as prototypes for other impurities and dopants that commonly occupy adatom sites. The three species also span a range of masses, which gives rise to spread in the frequencies of defect-related modes. Two different configurations were investigated: In the *X*_ad1_ geometry the *X* atoms reside directly above Mo/W, whereas in the *X*_ad2_ configuration the adatom sits above an empty “channel”.

While STM and STEM measurements enable measuring defect concentrations, this is still challenging—both due to the statistics involved and technical complexities such as concurrent beam damage in the case of electron microscopy.^50^ For MoS_2_ defect concentrations between 4 × 10^10^ and 5 × 10^13^ cm^–2^ have been measured.^13,14,17,19,20,51^ The data available for WS_2_ is sparser. Using STEM, a bulk defect concentration of 3.3 × 10^13^ cm^–2^ has been obtained,^15^ while STM measurements yielded a density of 2.3 × 10^10^ cm^–2^ for W and 4.5 × 10^11^ cm^–2^ for S vacancies.^41^ Given that electron irradiation is known to facilitate defect formation,^50^ it is plausible that the STEM values could be overestimates. The STM data, on the other hand, indicate that defect concentrations in WS_2_ might be lower than in MoS_2_, a suggestion that is supported by our analysis as will be discussed below.

**Figure 3:**
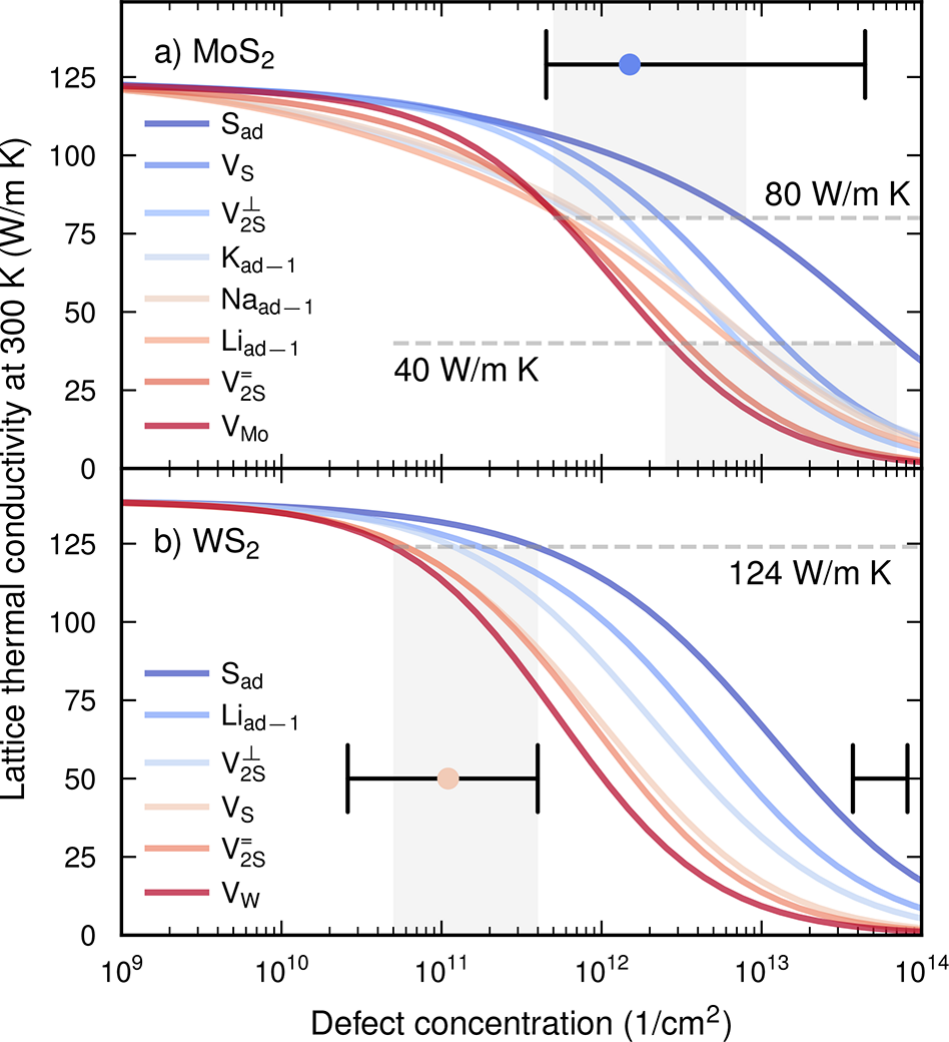
Lattice thermal conductivity at 300 K of (a) MoS_2_ and (b) WS_2_ as a function of concentration for different defects. Gray rectangles indicate the concentration range that approximately aligns with typical experimental values for the LTC. For MoS_2_ both an approximate upper (80 W K^–1^ m) and lower (40 W K^–1^ m) value are indicated. The horizontal bars represent the range of experimentally determined defect concentrations (see text for details). In the case of WS_2_ the data from refs (15) and (41) are shown separately. The colored circles indicate the S vacancy concentrations used in Figure 2a,b.

Because S vacancies are the most widely observed defect, we first focus our analysis on them. Increasing the concentration of S vacancies (and point defects generally; see the Supporting Information) leads to a drop of the LTC and more importantly a less pronounced temperature dependence (see Figure 2c for MoS_2_ and Figure S1 for WS_2_). This is expected because the rates of elastic scattering induced by defects are temperature independent, whereas inelastic three-phonon processes become more likely as the temperature increases. In MoS_2_ at room temperature the LTC varies between 135 W K^–1^ m^–1^ at a vacancy concentration of 10^10^ cm^–2^ and 9 W K^–1^ m^–1^ at 10^13^ cm^–2^ (Figure 3a). This range includes experimental measurements of the LTC, which span from 120 (ref (46)) to 35 W K^–1^ m^–1^ (ref (47)), which would be equivalent to defect densities of 7 × 10^10^ and 2 × 10^12^ cm^–2^, respectively.

Crucially, using typical defect densities from the middle of the experimentally observed range (see horizontal bars in Figure 3) yields good agreement with experimental data over the entire temperature range.^a^ For MoS_2_ and WS_2_ this suggests defect concentrations of approximately 1.5 × 10^12^ and 1.3 × 10^11^ cm^–2^, respectively. These concentrations thus differ by 1 order of magnitude, in line with the differences observed experimentally between these two materials (see above).

To date, we have considered only one type of defect, but obviously defects differ in their scattering efficacy. This can be illustrated by analyzing the variation of the LTC with defect concentration at a fixed temperature (300 K in Figure 3; see Figure S2 for more temperatures). In both MoS_2_ and WS_2_, S adatoms are the weakest and metal vacancies (V_Mo_, V_W_) are the strongest scatterers. However, as the concentrations needed to accomplish a certain LTC reduction only vary by about 1 order of magnitude (as indicated by the gray rectangles in Figure 3), the overall scattering efficiency is similar.

So far, we have shown that inclusion of defect scattering when predicting the LTC leads to near-quantitative agreement with experimental data without resorting to empirical parameters. It is now instructive to investigate the microscopic mechanism by which defects scatter phonons. Within the framework of Boltzmann transport theory, the LTC is predominantly determined by the phonon group velocities and lifetimes.

**Figure 4:**
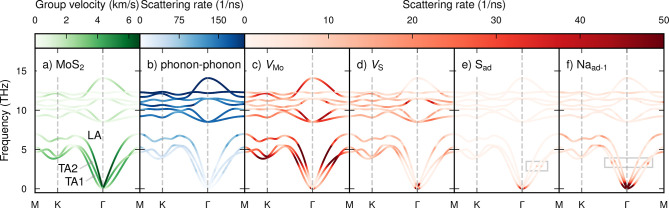
Phonon dispersion relations for MoS_2_ showing the (a) group velocities and (b) phonon–phonon scattering rates at 300 K as well as the defect scattering rates for (c) V_S_, (d) V_Mo_, (e) S_ad_, and (f) Na_ad–1_ defects at a concentration of 1 defect per 10^3^ unit cells (approximately 10^11^ cm^–2^). We chose this convention to enable a more direct comparison among defect types, independent of the number of atoms of each type in the unit cell.

It is apparent from the phonon dispersion (Figure 4a for MoS_2_; see also Figure S4 for WS_2_) that the acoustic branches are the main contributors to the thermal conductivity given their more significant group velocities and lower frequencies. As expected, the largest speed of sound is found in the longitudinal acoustic branch, followed by the transverse acoustic branches (labeled TA1 and TA2 in Figure 4a). The optical branches are only weakly dispersive; not only does this lead to lower group velocities but also enables more opportunities for the conservation of energy in three-phonon processes, leading to stronger anharmonic scattering, as seen in Figure 4b.

The overall magnitude of the phonon-defect rates follows the scattering efficiency observed via the LTC; that is, V_Mo_ scatters more strongly than V_S_, which scatters more strongly than S_ad_ (Figure 4c–e). All defects lead to scattering across the entire dispersion, but the lowermost acoustic modes close to the center of the Brillouin zone as well as the higher-frequency range of the acoustic branches are the most notably affected. The zero-frequency limit of the scattering rates deserves special mention because it deviates from the familiar Rayleigh power law found in 3D systems.^52^ In fact, the elastic scattering rates do not vanish as ω → 0 in quasi-2D systems due to the nonzero density of states in that limit.

There are signatures of resonant scattering for the S adatom as well as for the extrinsic adatoms (Figures 4e,f and S4) in the form of maxima in the scattering rates along the acoustic branches around 2.9 THz (S_ad_) and 3.3 THz (Na_ad–1_; marked by rectangles in Figure 4e,f). This effect is, however, less pronounced than in, e.g., B-doped SiC^11^ or for DX-center defects in GaAs^12^ and does not reach the level required to create a “superscatterer”.

## 4 Discussion

It is now instructive to compare our results and analysis with those of previous investigations.

Ding et al. employed an empirical interatomic potential and nonequilibrium molecular dynamics (MD) simulations with a thermal gradient to study the effect of Mo and S vacancies as well as S adatoms on the LTC in MoS_2_.^21^ They considered defect densities between 1.2 × 10^12^ and 1.2 × 10^13^ cm^–2^, at the upper end of the experimentally observed range (see below), and observed a reduction of the LTC by 35 to 60% at 300 K.

Peng et al., on the other hand, used Boltzmann transport theory with third-order FC from DFT calculations to assess the impact of S mono- and divacancies in MoS_2_ on the LTC.^22^ As a result of their approach, the defect concentration was fixed by the supercell size, yielding defect densities of 1.2 × 10^14^ cm^–2^ and above. They obtained a reduction for the in-plane LTC by up to 75% at 300 K. Moreover, this method treats scattering by those vacancies as inelastic, does so perturbatively, and assumes a perfectly periodic arrangement of the defects.

Combining Boltzmann transport theory with the **T**-matrix approach, Polanco and coauthors characterized the dependence of the LTC of MoS_2_ on both temperature and the concentration of S vacancies and adatoms.^23^ They concluded that the spread in the measured LTC is consistent with the experimentally observed variation in defect concentrations. According to their calculations, adatoms are much more effective phonon scatterers than vacancies, owing mainly to the fact that only the phonon-defect-scattering rates induced by the latter decay to zero at low frequencies.

Finally, using an empirical potential and equilibrium MD simulations, Gabourie et al. mapped out the dependence of the LTC at 300 K on the concentration of S vacancies in both suspended and supported MoS_2_.^24^ The simulation approach enabled them to access more realistic defect densities between 10^12^ and 5 × 10^13^ cm^–2^, and they found a 19% reduction already at the lower end of the concentration range considered, arriving at a similar conclusion as ref (23).

Our results deviate quantitatively and qualitatively from both the MD simulations by Ding et al.^21^ and from the BTE-based predictions by Polanco et al.^23^ Both of those references suggest that S adatoms are significantly stronger scatterers than the S vacancy and indeed that the former depresses the thermal conductivity to a much larger degree at a comparable concentration. However, ref (21) relies on a semiempirical potential and can be expected to provide limited quantitative insight into configurations where atomic environments are very different from those of the bulk, as is the case of the S adatom. Reference (23), on the other hand, employs a methodology much closer to our work and allows for a more detailed comparison. This reveals that the phonon–S adatom scattering rates reported by Polanco et al. have a finite zero-frequency limit, but the rates they report for the vacancy vanish as ω → 0. In the absence of a stated physical argument that could justify this contrasting behavior, the hypothesis of a numerical artifact cannot be ruled out. Specifically, the enforcement of the rotational acoustic sum rules, necessary for both the FC of the pristine system and those of the defect-laden structures, can be a significant challenge, and as stated above the Green’s function for the pristine system requires an extra postprocessing step to be able to deal with interstitial or adsorbed atoms. With regard to the acoustic sum rules, we are confident in our systematic use of the extensively tested hiphive package^36^ for all structures. Additionally, a comparison among the results for the sequence of alkaline adatoms Li_ad–1_, Na_ad–1_, and K_ad–1_ in Figure 3 serves as a qualitative test of our treatment of adatoms: The effect of the structurally and chemically similar Li_ad–1_ and Na_ad–1_ defects on the thermal conductivity is very much alike, but with the heavier Na_ad–1_ acting as a more intense scatterer, as expected. This reasoning also holds for K_ad–1_ in the high-concentration regime, where elastic scattering dominates. In this context, the effect of the sulfur adatom is perfectly reasonable for an element of its mass.

## 5 Conclusions

In this work, we have quantified the impact of point defect scattering on the LTC in MoS_2_ and WS_2_. When considering only phonon–phonon and isotope scattering, the computed LTC significantly overestimates the experimental values, especially at low temperatures.

Defects, particularly sulfur vacancies, play a vital role in mediating phonon scattering in these TMDs. Our study suggests that the typically high point-defect concentrations in these materials, which may reach and exceed 10^13^ cm^–2^, can substantially influence the lattice thermal transport. Calculations on a range of both intrinsic and extrinsic defects prevalent in these materials confirmed the S vacancy as the most dominant scattering center in terms of abundance and strength. Importantly, using defect concentrations consistent with those reported in experimental studies brings our LTC predictions into quantitative alignment with the measured data across the temperature range. This not only demonstrates the importance of point defects for understanding the thermal conduction in these materials but also suggests that variations in measured LTC values can be related to differences in the point defect distributions. Compared to earlier work, we find that both intrinsic and extrinsic adatoms are relatively weak scatterers, a difference that we attribute to the treatment of the translational and rotational acoustic sum rules. In the present work, these were enforced, which removes spurious contributions in the zero-frequency limit that can otherwise affect the results.

In summary, our work underscores the importance of defect-mediated phonon scattering in governing the thermal transport properties of MoS_2_ and WS_2_ and probably also in other transition metal dichalcogenides. This deeper understanding of the underlying mechanisms provides valuable insights into tailoring the thermal properties of these materials, paving the way for their potential applications in next-generation electronic and thermal management devices.

## Supporting Information Available

Table summarizing the parameters used for extraction of the second-order FC; Figures showing the LTC of WS_2_ in the presence of S vacancies, the LTC of MoS_2_ and WS_2_ as a function of defect concentration, the phonon dispersion relations for MoS_2_ and WS_2_ with defect scattering rates (PDF)

## Footnotes

a Unfortunately, to the best of our knowledge, there are no studies for which defect concentrations and LTC have been measured for the same sample.
