# Supplementary material for: Quantitative Predictions of the Thermal Conductivity in Transition Metal Dichalcogenides: Impact of Point Defects in MoS2 and WS2 Monolayers
Source: J Phys Chem C Nanomater Interfaces. 2024 Jan 18;128(4):1709–16. doi: 10.1021/acs.jpcc.3c06820 (PMC10839904; doi:10.1021/acs.jpcc.3c06820)
Supplement: Supplementary file 1 — jp3c06820_si_001.pdf [file jp3c06820_si_001.pdf]

Supplemental Material

Quantitative predictions of the thermal conductivity in transition metal dichalcogenides: The impact of point defects in MoS_2 and WS_2 monolayers

Srinivasan Mahendran[1], Jesús Carrete[2], Andreas Isacsson[1], Georg K. H. Madsen[2], and Paul Erhart[1,*]

[1] *Department of Physics, Chalmers University of Technology, SE-41296, Gothenburg, Sweden*
[2] *Institute of Materials Chemistry, TU Wien, A-1060 Vienna, Austria*
[*] *erhart@chalmers.se*

Contents

Supplemental Tables **S2**
S1. Computational parameters S2

Supplemental Figures **S3**
S1. Lattice thermal conductivity of WS_2 in the presence of S vacancies S3
S2. Lattice thermal conductivity of MoS_2 and WS_2 as a function of defect concentration . . . S3
S3. Phonon dispersion relations for MoS_2 with defect scattering rates S4
S4. Phonon dispersion relations for WS_2 with defect scattering rates S4

Supplemental References **S5**

Supplemental Tables

Table S1: **Computational parameters.** Summary of parameters used for extraction of the second-order FCs of MoS_2 defect supercells. N_{par}: Total number of parameters; $N_{non\text{-}zero}$: Number of non-zero parameters; RMSE: root-mean-square error over training (t) and validation (v) sets in meV $Å^{-1}$.

Defect	N_{par}	$N_{non\text{-}zero}$	RMSE(t)	RMSE(v)
S_{vac}	7933	5714	1.6	2.3
S_{divac}	23 367	4687	7.4	10.0
$S_{divac-z}$	4064	3254	4.3	5.1
S_{ad}	8043	6759	1.4	2.1
K_{ad-1}	8030	5783	2.2	3.1
K_{ad-2}	8028	4818	2.3	3.2
Li_{ad-1}	8054	6122	2.4	3.6
Li_{ad-2}	8052	6764	2.0	3.0
Mo_{vac}	4049	3888	1.8	2.2
Na_{ad-1}	8045	6440	1.9	2.8
Na_{ad-2}	8043	5475	1.9	2.7

Supplemental Figures

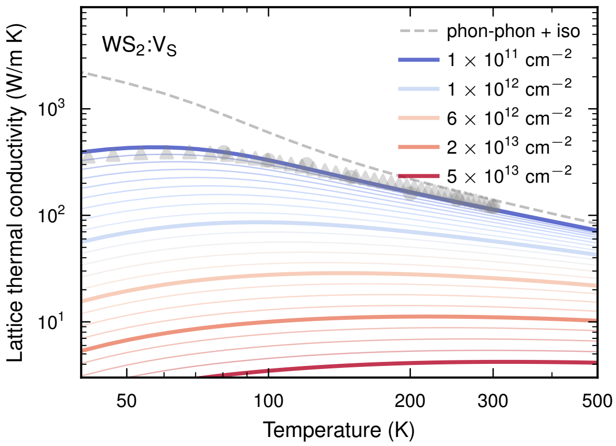

Figure S1: **Lattice thermal conductivity of WS_2 in the presence of S vacancies.** Temperature dependent experimental data from Jiang *et al.* (circles; Ref. 1) and Pisoni *et al.* (triangles; Ref. 2).

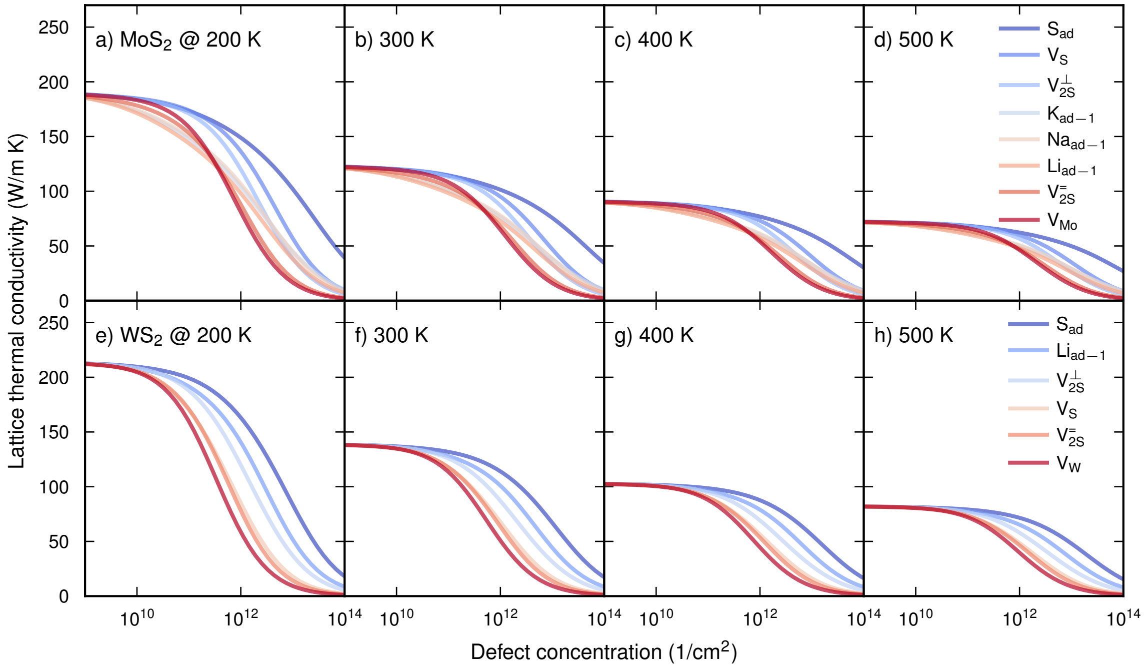

Figure S2: **Lattice thermal conductivity of MoS_2 and WS_2 as a function of defect concentration.**

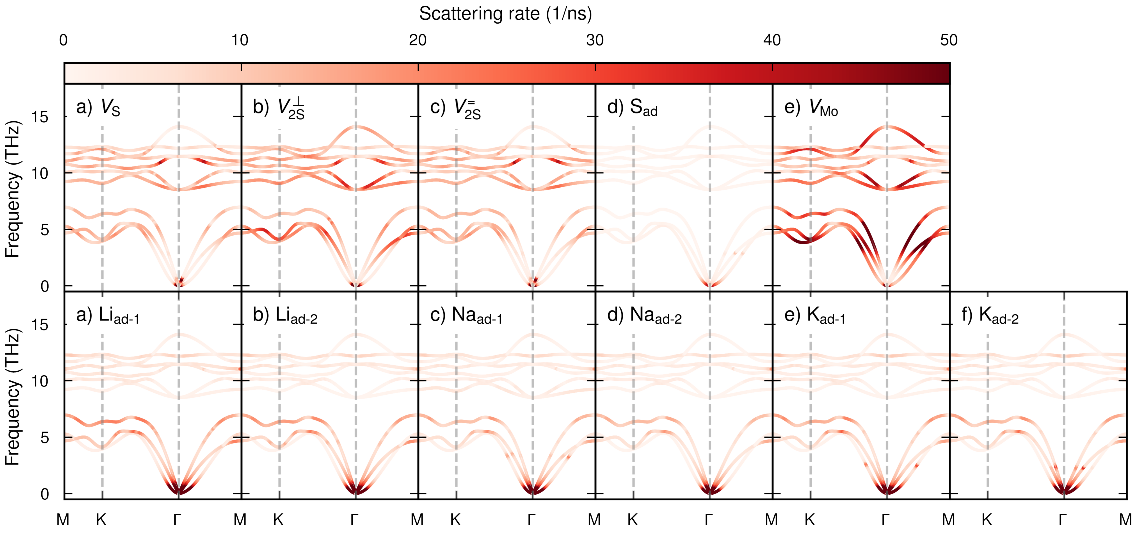

Figure S3: **Phonon dispersion relations for MoS_2 with defect scattering rates.** The scattering rates are shown for a concentration of 1 defect per 10^3 unit cells (approximately 10^{11} cm^{-2}).

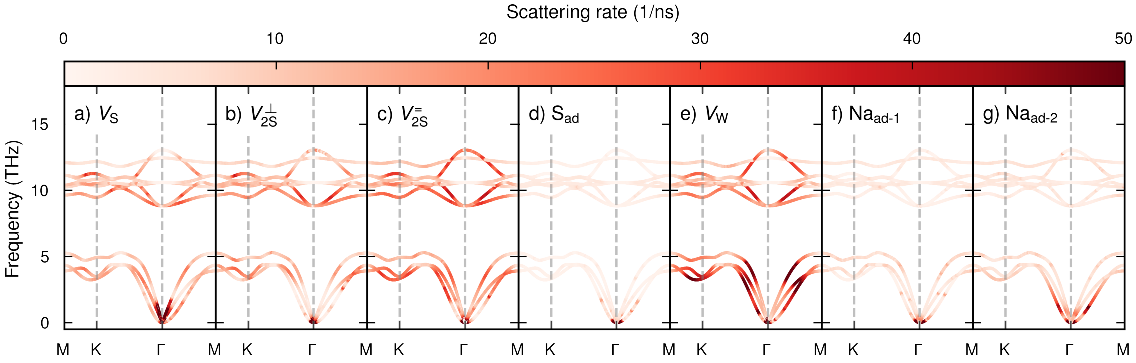

Figure S4: **Phonon dispersion relations for WS_2 with defect scattering rates.** The scattering rates are shown for a concentration of 1 defect per 10^3 unit cells (approximately 10^{11} cm^{-2}).

Supplemental References

(1) Jiang, P.; Qian, X.; Gu, X.; Yang, R. *Advanced Materials* **2017**, *29*, 1701068, DOI: 10.1002/adma.201701068.

(2) Pisoni, A.; Jacimovic, J.; Gaál, R.; Náfrádi, B.; Berger, H.; Révay, Z.; Forró, L. *Scripta Materialia* **2016**, *114*, 48–50, DOI: 10.1016/j.scriptamat.2015.11.028.
